# MafB Is Important for Pancreatic β-Cell Maintenance under a MafA-Deficient Condition

**DOI:** 10.1128/MCB.00080-19

**Published:** 2019-08-12

**Authors:** Gulibaikelamu Xiafukaiti, Shayida Maimaiti, Kiyohito Ogata, Akihiro Kuno, Takashi Kudo, Hossam H. Shawki, Hisashi Oishi, Satoru Takahashi

**Affiliations:** a Department of Anatomy and Embryology, Faculty of Medicine, University of Tsukuba, Ibaraki, Japan; b Laboratory Animal Resource Center, Faculty of Medicine, University of Tsukuba, Ibaraki, Japan; c School of Comprehensive Human Sciences, Doctoral Program in Biomedical Sciences, University of Tsukuba, Ibaraki, Japan; d Life Science Center, Tsukuba Advanced Research Alliance, University of Tsukuba, Ibaraki, Japan; e Transborder Medical Research Center, Faculty of Medicine, University of Tsukuba, Ibaraki, Japan; f International Institute for Integrative Sleep Medicine (WPI-IIIS), University of Tsukuba, Ibaraki, Japan; g Department of Comparative and Experimental Medicine, Nagoya City University Graduate School of Medical Sciences, Nagoya, Japan

**Keywords:** diabetes mellitus, MafA, MafB, pancreatic β cells

## Abstract

The pancreatic-islet-enriched transcription factors MafA and MafB have unique expression patterns in β cells in rodents. MafA is specifically expressed in β cells and is a key regulatory factor for maintaining adult β-cell function, whereas MafB plays an essential role in β-cell development during embryogenesis, and its expression in β cells gradually decreases and is restricted to α cells after birth in rodents. However, it was previously observed that MafB started to be reexpressed in insulin-positive (insulin^+^) β cells in MafA-deficient adult mice. To elucidate how MafB functions in the adult β cell under MafA-deficient conditions, we generated MafA and MafB double-knockout (A0B0) mice in which MafB was specifically deleted from β cells. As a result, the A0B0 mice became more vulnerable to diabetes under a high-fat diet (HFD) treatment, with impaired islet formation and a decreased number of insulin^+^ β cells because of increased β-cell apoptosis, indicating MafB can take part in the maintenance of adult β cells under certain pathological conditions.

## Abstract (precis)

The pancreatic-islet-enriched transcription factors MafA and MafB have unique expression patterns in β cells in rodents. MafA is specifically expressed in β cells and is a key regulatory factor for maintaining adult β-cell function, whereas MafB plays an essential role in β-cell development during embryogenesis, and its expression in β cells gradually decreases and is restricted to α cells after birth in rodents.

## INTRODUCTION

Diabetes mellitus (DM) is a serious chronic disease that causes the deaths of millions of people throughout the world. DM occurs when blood glucose homeostasis is impaired because either the pancreas is no longer producing insulin, which is called type 1 diabetes (T1DM), or the cells in the body have difficulty responding to insulin properly, which is called type 2 diabetes (T2DM) (1). Pancreatic β cells of the islets of Langerhans synthesize and secrete insulin in response to elevated blood glucose and nutrition to maintain blood glucose homeostasis (2). Therefore, studying the detailed mechanism of β-cell development, maintenance, and function is not only crucial for understanding the DM disease process but also important for providing advanced treatment of the disease.

MafA and MafB, members of the large Maf protein family, are known as islet-enriched transcription factors that play important roles in β-cell development, maintenance, and function (3). Their expression patterns in β cells are different from each other. In rodents, MafB starts to be expressed around embryonic day 10.5 (E10.5) in the pancreatic progenitor cells of both α and β cells. MafB expression in β cells gradually decreases and is restricted to α cells after birth. MafA, on the other hand, starts to be expressed at E13.5 and is expressed only in β cells throughout adulthood (4, 5). Several studies on MafA and MafB mutant mice have revealed that MafA is a fundamental regulatory factor for β-cell function. The phenotype of MafA-deficient adult mice showed reduced expression of insulin secretion genes, fasting hyperglycemia, and impaired glucose-stimulated insulin secretion (6, 7). MafB functions in β cells only in the embryonic stage, and MafB knockout (KO) embryos display decreased insulin-positive (insulin^+^) cell numbers; this phenomenon disappears soon after birth (8, 9), indicating that MafB plays important roles in β-cell development but not in β-cell function in the adult stage.

In a previous study, we observed that MafB is expressed in many insulin^+^ cells in MafA-deficient adult mice compared with wild-type (WT) mice (7). We have also found that MafA knockout, MafB heterozygote (A0B1) adult mice display more severe hyperglycemia than do A0 adult mice (10). Based on these results, we hypothesized that MafB can take part in the maintenance of adult β-cell function under pathological conditions. To investigate whether MafB takes part in adult β-cell activity under MafA-deficient conditions, we generated MafA, MafB double-knockout (A0B0) mice in which MafB was specifically deleted in β cells and compared its phenotype with those of A0 and WT mice.

## RESULTS

### The effect of MafB on insulin-producing cells gradually decreases after birth.

**FIG 1:**
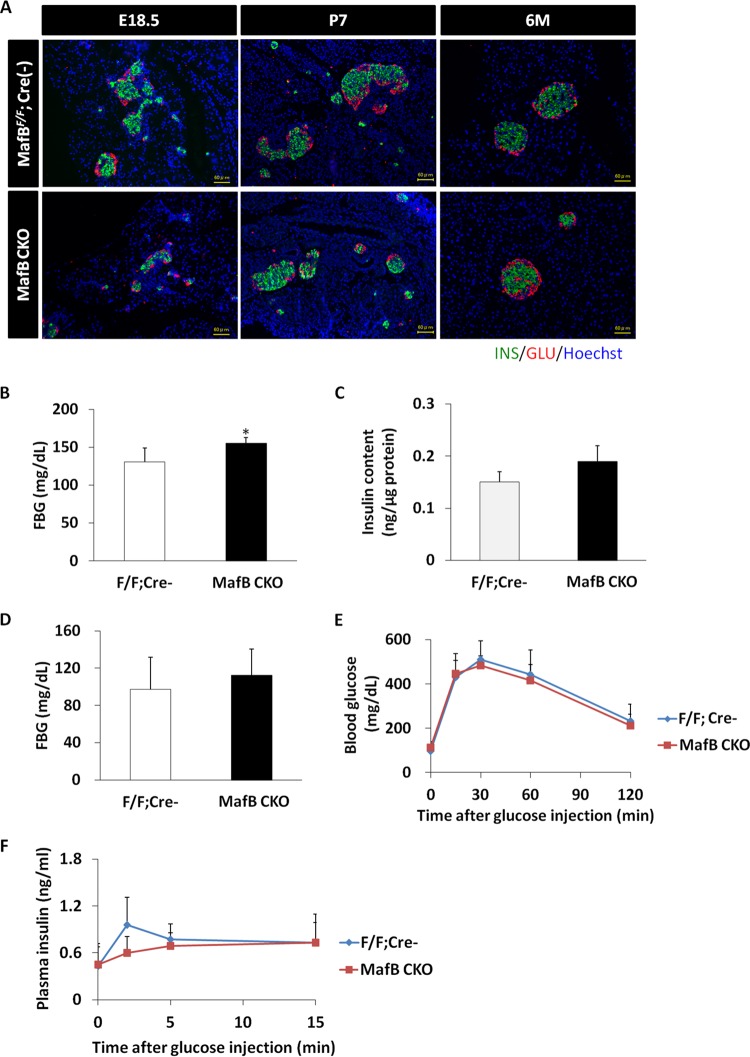
The effect of MafB on insulin-producing β cells does not last into adulthood. (A) Insulin (green) and glucagon (red) immunoreactivities in MafB^flox^/^flox^::Cre^−^ and MafB CKO mice at E18.5, P7, and 6 months of age (6M). (B) Fasting blood glucose (FBG) levels of mice at P7. Six or 7 mice from each genotype were used. F/F, MafB^flox^/^flox^. (C) Insulin contents of mice at P7. Four mice from each genotype were used. (D) Fasting blood glucose levels of mice at 6 months of age. Five mice from each genotype were used. (E) Glucose tolerance tests (ipGTT) after intraperitoneal loading with 2 g glucose/kg were performed on 6-month-old mice of the indicated genotypes following a 16-h fasting period. (F) *In vivo* GSIS tests after intraperitoneal loading with 3 g glucose/kg were performed on 6-month-old mice of the indicated genotypes following a 16-h fasting period. The data are from 5 male mice of each genotype. *, *P < *0.05. The error bars indicate SD.

It is well known that MafB regulates the transcription of genes needed for α-cell and β-cell production; thus, absence of MafB results in a reduction in α- and β-cell numbers during embryonic development (8, 9, 11, 12). To investigate MafB regulation of β cells alone, we generated MafB conditional-knockout (CKO) mice in which MafB was specifically deleted from β cells by Cre-loxP recombination. MafB deletion from β cells was confirmed by immunofluorescence staining at the embryonic stage (see Fig. S1A in the supplemental material). We analyzed the histology of the pancreatic islets of CKO mice both prenatally and postnatally. MafB deficiency caused a minor effect on insulin-positive-cell proliferation, since reduced expression of insulin^+^ cells in CKO mice was observed at E18.5. Furthermore, this change was reversed after birth, because MafB is no longer expressed in β cells during postnatal development under normal conditions (Fig. 1A).

To check whether the prenatal defect in β-cell development could affect glucose metabolism postnatally in CKO mice, we measured the fasting blood glucose level and insulin content in the neonatal stage. Although the fasting blood glucose level was slightly elevated in CKO mice at postnatal day 7 (P7) compared to the control group (control, 125 ± 18 mg/dl; MafB CKO, 156 ± 7 mg/dl), the insulin content did not show any significant change at that age (control, 0.15 ± 0.02 ng/μg; MafB CKO, 0.19 ± 0.03 ng/μg) (Fig. 1B and C). Furthermore, the fasting glucose level became comparable to that of the control group at a later stage, along with unchanged glucose tolerance or glucose-stimulated insulin secretion (Fig. 1D to F).

### A0B0 mice showed mildly impaired glucose tolerance compared to WT and A0B2 mice.

**FIG 2:**
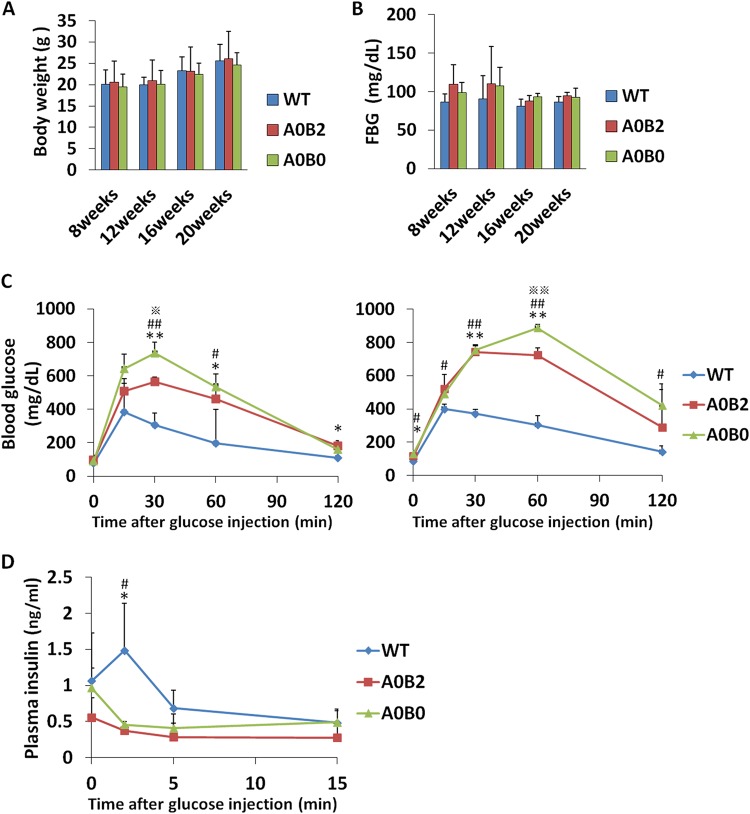
Deletion of MafB in β cells showing slightly impaired glucose tolerance in the A0B0 group compared to the WT and A0B2 groups. (A and B) Body weight and fasting blood glucose (FBG) levels of mice from different genotypes over 20 weeks. The data are from 3 to 6 male mice of each genotype. (C) Glucose tolerance tests (ipGTT) after intraperitoneal loading with 2 g glucose/kg were performed on 3- month-old (right) and 9-month-old (left) female mice of the indicated genotypes following a 16-h fasting period. (D) *In vivo* GSIS testing after intraperitoneal loading with 3 g glucose/kg was performed on 9-month-old female mice of the indicated genotypes following a 16-h fasting period. The data are from 3 or 4 female mice of each genotype. *, A0B2 and WT, *P < *0.05; **, A0B02 and WT, *P < *0.01; #, A0B0 and WT, *P < *0.05; ##, A0B0 and WT, *P < *0.01; ※, A0B2 and A0B0, *P < *0.05; ※※, A0B2 and A0B0, *P < *0.01. The error bars indicate SD.

In our previous study, we observed that MafB was reexpressed in adult β cells under MafA-deficient conditions (7). To elucidate the role of MafB in β cells postnatally in the absence of MafA, we generated A0B0 mice by mating the MafB CKO mice with the MafA-null knockout mice (A0B2). Body weights and fasting blood glucose levels were comparable within each group of mice (Fig. 2A and B). Next, we compared the glucose clearance abilities of WT, A0B2, and A0B0 mice by an intraperitoneal glucose tolerance test (ipGTT). Blood glucose levels were rapidly increased in both A0B0 and A0B2 mice after glucose administration and were maintained at high levels compared with that of WT mice for at least 2 h. A0B0 mice showed a significantly elevated blood glucose level relative to that of A0B2 mice at 30 min (A0B0, 693 ± 119 mg/dl; A0B2, 578 ± 27 mg/dl; *P < *0.05) (Fig. 2C). A0B0 mice became more intolerant of glucose 6 months later, as their blood glucose levels reached nearly 900 mg/dl 1 h after glucose administration and were still at a high level (422 ± 130 mg/dl) at 2 h. To investigate the mechanism of this glucose intolerance, we analyzed the insulin secretion of the mice in response to glucose stimulation. Plasma insulin levels in each mouse group were not significantly different from each other before glucose stimulation. Both A0B2 and A0B0 mice failed to respond to an elevated glucose level, since there was no acute insulin secretion after glucose administration. However, the insulin level of the A0B0 mice was comparable to that of the A0B2 mice (A0B2 versus A0B0, 0 min, 0.56 ± 0.27 ng/μg versus 0.96 ± 0.5 ng/μg; 2 min, 0.37 ± 0.04 ng/μg versus 0.46 ± 0.04 ng/μg; 5 min, 0.28 ± 0.20 ng/μg versus 0.41 ± 0.02 ng/μg; 15 min, 0.27 ± 0.16 ng/μg versus 0.49 ± 0.02 ng/μg) (Fig. 2D). These results indicate the glucose tolerance capacity was impaired in a mild range in A0B0 mice, while their insulin secretion capacity in response to glucose stimulation was unchanged compared to A0B2 mice.

### Impaired islet structures were observed in both A0B2 and A0B0 pancreases but more significantly in A0B0 mice.

**FIG 3:**
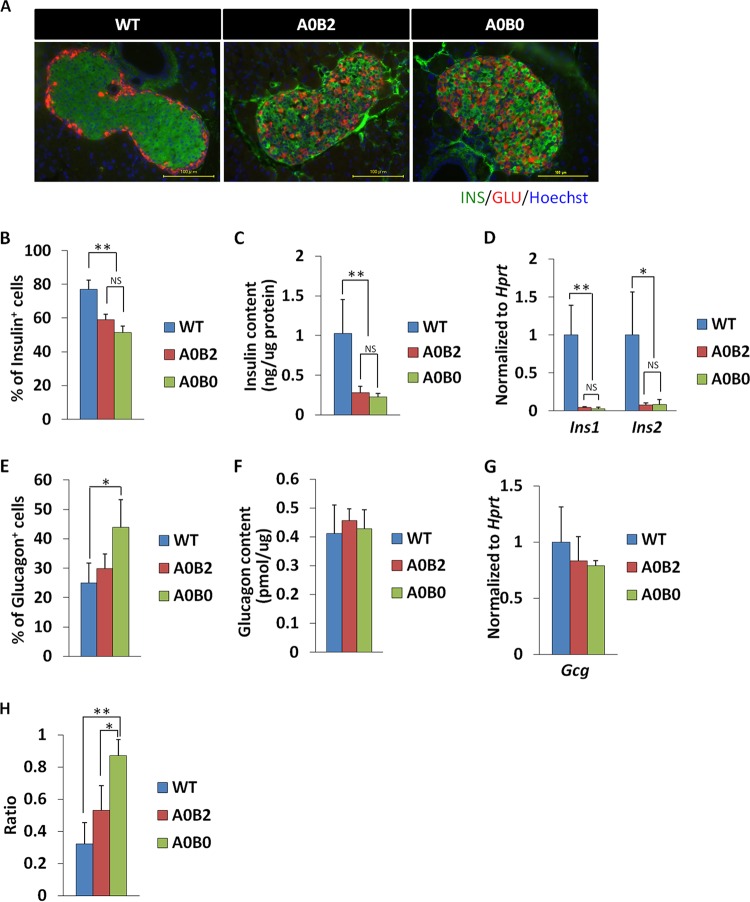
Impaired islet structure is more significant in the A0B0 pancreas than in the WT and A0B2 pancreas. (A) Insulin (green) and glucagon (red) immunoreactivities in mouse pancreatic islets from 9-month-old female mice of each genotype. (B) Insulin-positive cell number/total islet cell number ratio in pancreatic islets of each mice group. (C) Insulin contents of mice from different genotypes. The data are from 3 to 9 males of each genotype at 9 months of age. (D) *Ins1* and *Ins2* gene expression in islets from each genotype. The amount of each transcript was normalized to the amount of the *Hprt* transcript. The expression levels of the *Ins1* and *Ins2* genes in the WT were set as 1. The data are from 3 or 4 female mice of each genotype at 9 months. (E) Glucagon-positive cell number/total islet cell number ratio in pancreatic islets of each genotype. (F) Glucagon contents of mice from different genotypes. The data are from 3 to 9 males of each genotype at 9 months. (G) *Gcg* gene expression of islets from each genotype. The amount of *glucagon* transcript was normalized by the amount of the *Hprt* transcript. (H) Glucagon-positive cell number/insulin-positive cell number ratio in pancreatic islets of each genotype. The data are from 3 or 4 female mice of each genotype at 9 months. *, *P* < 0.05; **, *P* < 0.01; NS, not significant. The error bars indicate SD.

Since A0B0 mice displayed more impaired glucose tolerance, we next analyzed the pancreatic islets of A0B0 mice histologically and compared them with those of WT and A0B2 mice. Using anti-insulin and anti-glucagon antibodies, the numbers and percentages of insulin- and glucagon-positive cells were measured (Fig. 3A, B, and E). There was a significant reduction in insulin-expressing cell numbers in the pancreatic islets of both A0B2 and A0B0 mice compared to WT mice. However, the difference between the A0B2 and A0B0 mouse groups was insignificant (Fig. 3A and B). The quantitative PCR results from the isolated pancreatic islets showed that the amounts of mouse insulin genes *Ins1* and *Ins2* were reduced in A0B2 and A0B0 islets in comparison to WT islets, while they were comparable within the groups (Fig. 3D). Whole pancreatic insulin content measurements also showed the same results (Fig. 3C).

The number of glucagon-expressing cells, on the other hand, showed a significant increase in A0B0 islets compared to that in WT islets (WT, 24.97 ± 6.7; A0B0, 43.9 ± 9.4) (Fig. 3E). The ratio of glucagon-expressing cells to insulin-expressing cells in A0B0 islets also increased correspondingly and showed a significant difference from WT and A0B2 islets (WT, 0.32 ± 0.13; A0B2, 0.53 ± 0.16; A0B0, 0.87 ± 0.10) (Fig. 3H). These results indicate that structural destruction was more prominent in the A0B0 mouse group than in the other groups. However, the relative mRNA expression of glucagon and the whole pancreatic glucagon content were comparable among the groups (Fig. 3G and F, respectively).

### A0B0 mice developed diabetes 5 months after high-fat-diet (HFD) treatment.

The phenotype of MafA knockout mice in our study, which had a C57BL/6J background, was relatively mild compared with that of mice with an ICR background. In mice from the ICR strain, a deficiency of MafA contributed to fasting hyperglycemia at an early stage of adulthood (6). However, with a C57BL/6J background, the fasting blood glucose level of the MafA knockout mice was comparable to that of WT mice even at a later stage of adulthood (7). In addition, although MafA knockout mice from the ICR strain developed diabetes as a result of long-term fasting hyperglycemia (6), MafA knockout mice from the C57BL/6J strain displayed only impaired blood glucose tolerance and did not show any other severe phenotype. Since the deletion of MafA from the C57BL/6J mice was successful (see Fig. S1A and B), we thought that, in the C57BL/6J mouse strain, deficiency of MafA alone was not enough to induce the impact of MafB on adult β cells. Hence, in order to induce a more pathological condition, we treated the mice with an HFD to develop a diabetic phenotype.

**FIG 4:**
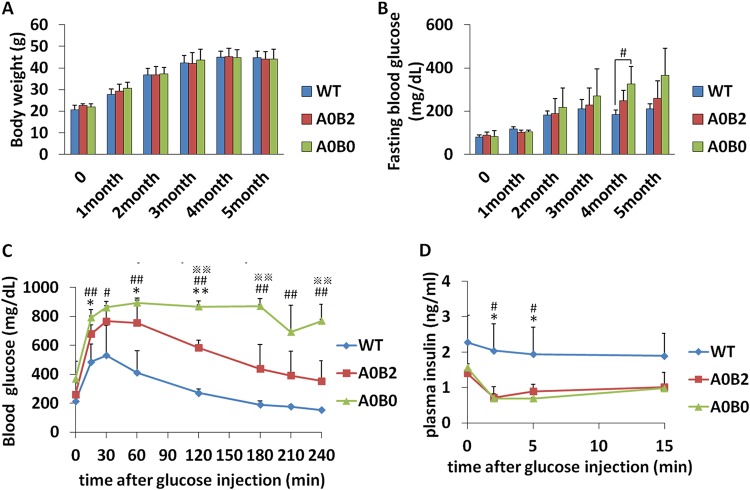
Blood glucose tolerance is severely impaired in A0B0 mice under HFD treatment. (A and B) Body weights and fasting blood glucose levels from mice with different genotypes during the 5-month HFD feeding period. The mice were fed an HFD from 8 weeks of age. The data are from 3 or 4 male mice of each genotype. (C) Glucose tolerance tests (ipGTT) after intraperitoneal loading with 2 g glucose/kg were performed on male mice of the indicated genotypes following a 16-h fast after 5 months of HFD treatment. (D) An *in vivo* glucose-stimulated insulin secretion (GSIS) test after intraperitoneal loading with 3 g glucose/kg was performed on 5-month-HFD-treated male mice of the indicated genotypes following a 16-h fasting period. The data are from 3 or 4 male mice of each genotype. *, A0B2 and WT, *P < *0.05; **, A0B02 and WT, *P < *0.01; #, A0B0 and WT, *P < *0.05; ##, A0B0 and WT, *P < *0.01; ※, A0B2 and A0B0, *P < *0.05; ※※, A0B2 and A0B0, *P < *0.01. The error bars indicate SD.

Body weight and fasting blood glucose levels were measured before and after the HFD treatment. Feeding an HFD increased the body weights of all the groups to the same degree (Fig. 4A). Fasting blood glucose levels of A0B0 mice tended to be higher and were elevated significantly compared to the other groups 4 months after initiating an HFD (Fig. 4B). Glucose tolerance was severely impaired in the A0B0 mouse group, as the blood glucose level remained significantly higher even at 4 h post-glucose administration (800 mg/dl) (Fig. 4C).

In addition, A0B0 mice started to show symptoms of diabetes, such as frequent urination with a high urine glucose level (data not shown). Two of them died because of this severe condition.

### Total islet number is decreased and islet size is not properly maintained in HFD-treated A0B0 mice.

**FIG 5:**
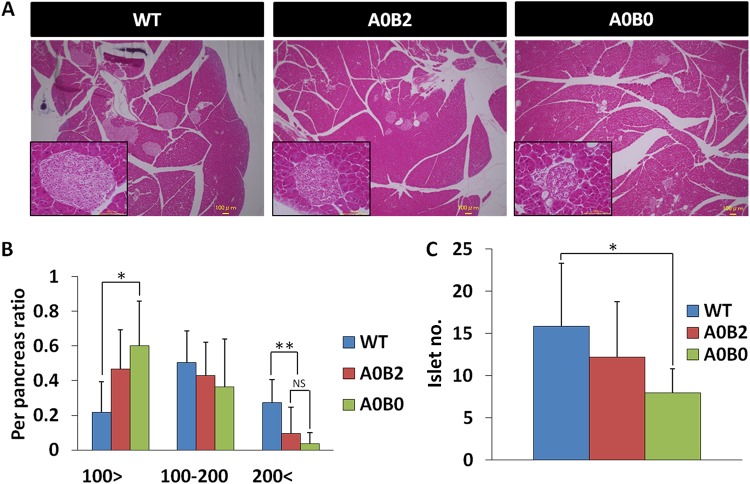
A0B0 mice with an HFD had decreased total islet numbers, and the islets failed to properly expand. (A) Hematoxylin-and-eosin staining of mice from different genotypes after 5 months of HFD feeding. (B) Morphometric analysis of islet diameters in pancreases from each mouse group after 5 months of HFD feeding. (C) Total islet numbers in pancreases from each mouse group. The data are from 3 or 4 mice of each genotype. *, *P* < 0.05; **, *P* < 0.01; NS, not significant. The error bars indicate SD.

We next analyzed the pancreatic islets histologically by hematoxylin-and-eosin (H&E) staining (Fig. 5A). The numbers and sizes of the islets were quantified in WT, A0B2, and A0B0 mice. Islets were divided into 3 groups according to their size—(i) small islets (less than 100 μm in diameter), (ii) medium-size islets (100 μm to 200 μm in diameter), and (iii) large islets (more than 200 μm in diameter)—and their proportions in each mouse group were calculated. The percentages of large islets were decreased significantly in the A0B2 and A0B0 groups compared to that in the WT group (WT, 27% ± 5.7%; A0B2, 11% ± 4.2%; A0B0, 4% ± 0.6%), while they were comparable to each other. Middle-sized islets, on the other hand, were almost equally distributed in all 3 groups. Interestingly, we found that the percentage of small islets was significantly higher in the A0B0 group than in the WT group, while it remained comparable to that in the A0B2 mouse group (WT, 22% ± 9.4%; A0B2, 45% ± 6.1%; A0B0, 60% ± 6.5%) (Fig. 5B). The total number of islets in the A0B0 mouse group dropped significantly compared to the WT mice (WT, 16 ± 7.5; A0B0, 8 ± 2.9) (Fig. 5C). To sum up, small islets made up a larger proportion and large islets made up a smaller proportion in the A0B0 mouse group than in any other group, while the total number of islets was also significantly decreased in that group.

### Insulin-positive cell numbers are significantly decreased in HFD A0B0 mice.

**FIG 6:**
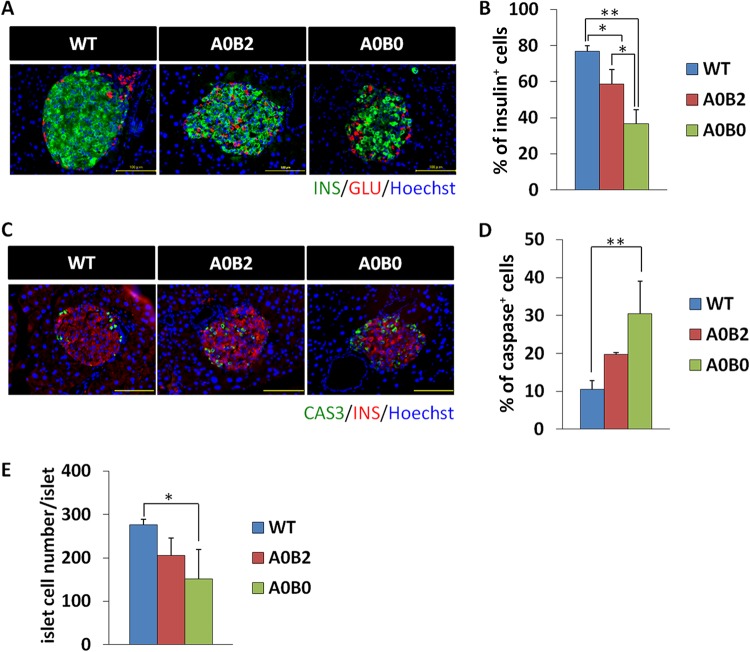
Insulin production is severely impaired in islets of A0B0 mice compared to WT and A0B2 mice. (A) Insulin (green) and glucagon (red) immunoreactivities in mouse pancreatic islets from each genotype after HFD treatment. (B) Insulin-positive cell number/total islet cell number ratio in pancreatic islets of each mouse group. (C) Cleaved caspase 3 (green) and insulin (red) immunoreactivities in mouse pancreatic islets from each genotype after HFD treatment. Scale bars, 100 μm. (D) Cleaved caspase 3-positive cell number/total islet cell number ratio in pancreatic islets of each mouse group. (E) Total islet cell number per pancreatic islet of each mouse group. The data are from 3 or 4 mice of each genotype. *, *P* < 0.05; **, *P* < 0.01. The error bars indicate SD.

We next analyzed the islet structure of HFD-treated mice by staining the islets with insulin and glucagon antibodies. Insulin-positive cells showed a drastic reduction in number in the A0B0 group compared to those in the WT and A0B2 groups (the percentages of insulin^+^ cells per islet were as follows: WT, 76% ± 3.2%; A0B2, 59% ± 8.2%; A0B0, 37% ± 7.9%) (Fig. 6A and B). To determine if the reduced number of insulin^+^ cells in A0B0 mice was due to decreased cell proliferation and/or increased cell apoptosis, we stained the islets with Ki67 (a proliferative-cell marker) and cleaved caspase 3 (an apoptotic-cell marker) antibodies, respectively. Ki67 signaling could be observed at E18.5 in insulin^+^ cells (see Fig. S4A in the supplemental material), as the cells are highly proliferative during embryonic development. However, there were no detectable Ki67^+^ cells in the islets of any mouse group with 5-month HFD treatment (see Fig. S4A). Cleaved caspase 3^+^ cells, on the other hand, were significantly increased by proportion in the islets of the A0B0 group compared to those of the WT group (the percentages of cleaved caspase 3^+^ cells per islet were as follows: WT, 11% ± 2.2%; A0B2, 19% ± 0.5%; A0B0, 31% ± 8.5%) (Fig. 6C and D), indicating cell apoptosis is more highly induced in A0B0 islets. Finally, total islet cell numbers per islet in the A0B0 mice showed a significant reduction compared to those in the WT mice, while they were comparable to those in the A0B2 mice (Fig. 6E). This might due to the increased number of small islets in the A0B0 mouse group (Fig. 5B).

## DISCUSSION

In this study, we generated and analyzed mice with single and compound knockouts of MafA and MafB to determine the role of MafB in adult β cells under MafA knockout conditions. Several main observations on pancreatic function and islet morphology are reported here. First, A0B0 adult mice displayed more severely impaired glucose tolerance than A0B2 mice. This phenomenon was further aggravated with HFD treatment, which caused diabetes in the A0B0 mice. Second, deficiency of MafA has a destructive effect on normal islet structure, and moreover, the abnormality becomes more significant with the deletion of MafB. Third, a notable reduction in islet cell numbers, together with an increase of β-cell apoptosis, was detected in HFD-fed A0B0 mice. Therefore, our findings provide evidence for a functional role of MafB in maintaining mature β-cell features under some specific pathological conditions.

As a key regulator for α-cell and β-cell development and mature α-cell function, deletion of MafB specifically from β cells only delayed β-cell development, as expected (Fig. 1A). During embryogenesis, MafB is not only required for the transcription of *Ins1* and *Ins2*, it also important for maintaining a normal expression level of key β-cell genes, such as *Pdx1* and *Glut2*, since deletion of MafB resulted in a reduction of expression of the above-mentioned genes (11, 12), thereby affecting normal β-cell development. MafB’s effect on β cells gradually decreases after birth (Fig. 1C to E), along with increased expression of MafA in β cells, due to the MafB^+^-to-MafA^+^ transition being crucial for β-cell functional maturation (5).

Deletion of MafA, on the other hand, resulted in reduced *Ins1*, *Ins 2*, and *Pdx1* transcription in adult mice, which further led to impaired glucose tolerance and glucose-stimulated insulin secretion. These results are consistent with previous studies demonstrating that MafA regulates glucose-stimulated insulin secretion by promoting transcription of *Insulin* and other genes related to β-cell genes (6, 13–18). Interestingly, the fasting blood glucose concentration was sustained at a normal level as a control in this study, while we found in our previous study (6) that MafA-deficient mice developed diabetes because of hyperglycemia. Since the MafA KO mice were based on the C57BL/6J strain in the present study while Zhang et al. used the ICR strain, they were generated from different genetic backgrounds, and strain differences could explain the phenotypic variations. Almost the same result was reported by Nishimura et al. (7).

Deletion of MafA and MafB together aggravated the metabolic phenotype of MafA single-knockout mice. More impaired glucose intolerance in A0B0 mice than in A0B2 mice was observed under normal diet conditions, which was severely aggravated by HFD feeding and led to diabetes mellitus in the double-knockout mice. The undermined glucose tolerance was due to either normal insulin production being affected, which results in reduced insulin content, or normal insulin release in response to an elevated blood glucose level being impaired. Neither the whole pancreatic insulin content nor the glucose-stimulated insulin secretion showed significant differences between the A0B2 and A0B0 mice under normal diet conditions. Interestingly, the α-cell/β-cell ratio became remarkably higher in the A0B0 islets than in the A0B2 islets. Impaired islet structure is one of the significant phenotypes of MafA-deficient mice (6), but the molecular mechanisms leading to this structural abnormality have not been clarified. Since this abnormality became more remarkable in the A0B0 group, we assumed it could explain the more impaired glucose tolerance in A0B0 mice than in A0B2 mice under normal diet conditions. Interestingly, Cyphert et al. showed that expressing the MafB homodimer in MafA^Δβ^ (with MafA specifically deleted in β cells) mice resulted in the same phenotype as MafA^Δβ^ mice with impaired islet structure, and their glucose tolerance was not rescued by MafB expression (19). Taken together with our results, this shows that MafB has other unique properties rather than just acting as a compensatory factor for MafA to maintain adult β-cell function.

Since conversions between α and β cells were reported in previous studies (20, 21), it is possible that MafB could be deleted from β-cell-converted α cells or vice versa in the present study. However, there were no double-positive Pdx1 or glucagon cells in the islets of any mouse group (see Fig. S3A in the supplemental material), indicating conversions of glucagon-producing cells to insulin-producing cells had not occurred at a noticeable level. This was further confirmed by Pdx1/Arx staining (see Fig. S3B). In addition, all the insulin-positive cells were Pdx1 positive in the islets of all three groups (see Fig. S3C). Taken together, these results suggest that there is no obvious cell conversion in A0B0 islets. As downstream transcription factors for islet endocrine cell differentiation, deletion of MafA and MafB may have a small effect on cell fate determination.

Realizing that, on a C57BL/6J background, a deficiency of MafA alone was not sufficient to show the possible role of MafB in maintaining adult β-cell function, we implemented HFD treatment for mice to induce obesity and obtain a more severe phenotype. As a result, insulin^+^ cell numbers showed a dramatic reduction in A0B0 islets, while glucose-stimulated insulin secretion levels were comparable between the A0B2 and A0B0 groups (Fig. 4D), indicating that critically damaged insulin production ability of β cells could be a reason for A0B0 mice to become extremely glucose intolerant and to develop diabetes. Nevertheless, their insulin content needs to be measured to confirm this conclusion.

In insulin resistance disorders caused by adiposity or gestation, the β-cell mass increases to adapt to the body’s increased requirement for insulin, which results in enlarged islets and increased islet numbers (22–26). Generally, the β-cell mass is regulated by β-cell proliferation and β-cell apoptosis (27). Previous studies found that MafB expression is induced in proliferative β cells during pregnancy and in obesity (28, 29), suggesting MafB may play a unique role in β-cell adaptation under certain metabolic stresses that demand more insulin production. Deficiency of MafA under HFD conditions is harmful enough to cause insulin insensitivity in the body. We speculate that removing MafB from β cells further inhibits the β-cell mass from expending by increasing islet numbers and islet size. The drastic reduction in insulin^+^ β cells in A0B0 mice supports our hypothesis. However, we failed to detect and compare the β-cell proliferation in the islets of WT, A0B2, and A0B0 mice with that in mice undergoing 5-month HFD treatment (Fig. S4A). Adaptive β-cell proliferation has been reported to start within the first week of HFD treatment (30). Therefore, it might be already completed by 5 months after the beginning of HFD treatment. That is why we could not detect β-cell proliferation at this period. On the other hand, we clearly detected an increase of β-cell apoptosis in A0B0 islets (Fig. 6C and D). Although this was an unexpected result, it may be reasonable, because MafB is known to be a regulator to prevent apoptosis in macrophages under specific conditions, as we reported previously (31). Therefore, this finding provides a whole new perspective on MafB function in pancreatic β cells.

Finally, unlike rodents, where MafB expression in β cells declines postnatally, MAFB is expressed in human β cells along with MAFA throughout adulthood (8, 32), implying its importance in maintaining mature human β-cell function. To mimic the human condition in mice, Cyphert et al. overexpressed MafB in mouse adult β cells using MafA transcriptional-control sequences. However, no overt metabolic effect was observed upon coexpression of MafA and MafB, indicating MafA-MafB heterodimers can function like MafA homodimers to regulate human adult β cells (19). Notably, overexpressing MafB in human β-cell lines (βTC3 cells) resulted in increased cell proliferation by upregulating important cell cycle regulators, like cyclin D2 and cyclin B (28). MafB expression has also been found to be acutely decreased in type 2 diabetic humans (33). Taken together, the data show that reduced expression of MafB in human islets may impede the proliferation of β cells that is normally triggered by increased metabolic stress and stimulate apoptosis, as we demonstrate in this paper, leading to diabetes mellitus. The detailed molecular mechanisms need further investigation.

To sum up, A0B0 mice became more susceptible to diabetes under HFD conditions, with impaired islet morphology and decreased insulin-expressing cell numbers because of apoptosis, indicating MafB is important for pancreatic β-cell maintenance under specific pathological conditions.

## MATERIALS AND METHODS

### Mice.

All experimental mice were maintained under specific-pathogen-free conditions in the Laboratory Animal Resource Center at the University of Tsukuba, Ibaraki, Japan. All experiments were performed according to the relevant Japanese and institutional laws and guidelines and approved by the University of Tsukuba Animal Ethics Committee (authorization number 14-189). The C57BL/6J mouse strain from the Jackson Laboratory was used in this study.

### Generation of A0B0 mice.

MafA-deficient (A0) mice were generated as previously described (6). Pancreatic-β-cell-specific deletion mutants of MafB (B0) were generated by crossing MafB^flox/flox^ (34, 35) mice with Ins1-Cre mice (36), which produced Cre in pancreatic β cells. A0B0 mice were generated by crossing A0 mice with B0 mice.

### High-fat diet.

MafA single-knockout (A0B2) and MafA and MafB double-knockout (A0B0) mice and their WT littermates were fed an HFD consisting of 62.2% fat, 19.6% carbohydrate, and 18.2% protein content on a caloric basis (Oriental Yeast, Japan) from 6 to 8 weeks of age.

### Immunohistochemistry.

Pancreatic tissues obtained from WT, A0B2, and A0B0 mice were fixed in 4% paraformaldehyde overnight at room temperature and embedded in paraffin. The sections were then deparaffinized in xylene (Fujifilm, Japan) 3 times for 5 min each, followed by rehydrating in graded ethanol (100% [5 min] and 95%, 85%, 70%, and 50% [2 min each]). They were placed in tap water for 5 min and then in phosphate-buffered saline (PBS) for 5 min (3 times) and blocked with PBS containing 10% goat serum with 0.03% preservative sodium azide for 1 h at room temperature. Then, they were incubated with guinea pig anti-insulin (1:100; ab7842; Abcam), rabbit antiglucagon (1:500; 2760; Cell Signaling), rabbit anti-MafA (1:100; Bethyl), rabbit anti-Pdx1(1:500; Chemicon), rabbit anti-Arx (1:250; a generous gift from Kunio Kitamura and Kenichirou Morohashi, Kyushu University, Japan), rabbit anti-MafB (1:100; IHC-00351; Bethyl), rabbit anti-Ki67 (1:200; Novocastra), and rabbit anti-cleaved caspase 3 (D175; Cell Signaling) overnight at 4°C. After rinsing, the slides were incubated with Alexa Fluor-conjugated secondary antibodies (anti-guinea pig antibody–Alexa Fluor 594 for insulin; anti-rabbit antibody–Alexa Fluor 488 for glucagon and cleaved caspase 3; and anti-rabbit antibody–Alexa Fluor 594 for MafA, Pdx1, Arx, Ki67, and MafB; 1:1,000; Life Technologies) for 2 h at room temperature. Tissue specimens were mounted with Fluoromount (Diagnostic Biosystem). All images were acquired on a fluorescence microscope (Biorevo BZ-9000; Keyence).

### Cell counting.

Following immunofluorescence staining, the different cell types in each islet were manually counted in the islet microscopy images. For each cell type, 10 to 30 representative islets from 3 or 4 mice per group were counted. To calculate the fraction of hormone^+^ cells within the islets, the number of hormone^+^ cells per islet was manually determined using ImageJ software and divided by the total number of Hoechst^+^ nuclei from the same islet.

### Islet counting and islet size measurement.

Pancreatic tissue 5-μm paraffin sections obtained from WT, A0B2, and A0B0 mice were stained by routine H&E staining. Islets in each section were manually counted in the islet microscopy images. Islet diameters were measured using ImageJ software.

### Pancreatic insulin and glucagon content measurement.

Whole pancreases obtained from the WT, A0B2, and A0B0 mice were weighed and chopped into small pieces in 6 ml ice-cold acid-ethanol (1.5% HCl in 75% ethanol). The tissues were then sonicated for 30 s (duty cycle, 20%; output control, 20) and stored overnight at 4°C, followed by a second round of sonication the next day. The supernatants were collected after centrifuging the samples at 2,400 rpm for 30 min at 4°C. The total protein concentration was measured with a Coomassie protein assay reagent (Thermo Scientific). Insulin and glucagon contents were measured by enzyme-linked immunosorbent assay (ELISA) (Morinaga mouse insulin ELISA kit [M1102]; Mercodia glucagon 10-μl ELISA kit [10-1281-OD]). Each pancreatic content was normalized by the total protein concentration per sample.

### Intraperitoneal glucose tolerance test.

Mice were fasted for 16 h and given glucose (2 g/kg body weight) by intraperitoneal injection. Blood samples were obtained from the tail vein at the indicated times (0, 15, 30, 60, and 120 min after injection) and then measured with a glucometer (Terumo, Japan).

### *In vivo* glucose-stimulated insulin secretion (GSIS) experiment.

Mice were fasted for 16 h and given glucose (3 g/kg) by intraperitoneal injection. Blood was collected into heparinized tubes from the facial vein at 2 min after glucose injection. Plasma insulin levels were measured with an insulin ELISA kit (Morinaga mouse insulin ELISA kit).

### Isolation of pancreatic islets.

Pancreatic islets were isolated from 9-month-old female WT, A0B2, and A0B0 mice as previously described (9).

### Quantitative real-time PCR.

Total RNA was extracted in Isogen (Nippon Gene) from isolated pancreatic islets of female WT, A0B2, and A0B0 mice. cDNA was synthesized according to the protocol of the QuantiTect reverse transcription kit (Qiagen). Real-time PCRs were performed in duplicate on a Thermal Cycler Dyce real-time system (Takara). The expression levels of all the target genes were normalized to that of *Hprt*. The primers used in this study are listed in Table 1.

**TABLE 1:** Primer sequences for real-time quantitative PCR

Gene	Orientation^*a*^	Sequence (5′→3′)
*Mafa*	F	GCTGCTGCACCCGCTTGAAG
	R	AGGCCACCACGTGCGCTTGG
*Ins1*	F	CCAGCTATAATCAGAGACCA
	R	GGGCCTTAGTTGCAGTAGTT
*Ins2*	F	AGGAAGCCTATCTTCCAGGT
	R	ATTCATTGCAGAGGGGTAGG
*Gcg*	F	AGGGACCTTTACCAGTGATGT
	R	AATGGCGACTTCTTTGGGAA
*Hprt*	F	TTGTTGTTGGATATGCCCTTGACTA
	R	AGGCAGATGGCCACAGGACTA

a F, forward; R, reverse.

### Statistical analysis.

Results were expressed as means and standard deviations (SD). Statistical comparisons between two groups were made using Student's *t* test, and comparisons among three groups were made using one-way analysis of variance (ANOVA) with *post hoc* Tukey honestly significant difference (HSD) tests. A statistically significant difference was defined as a *P* value of *<*0.05.

## Supplementary Material

Supplemental file 1

Supplemental file 2

Supplemental file 3

Supplemental file 4

Supplemental file 5
