# Supplementary figures and images for: MafB Is Important for Pancreatic β-Cell Maintenance under a MafA-Deficient Condition
Source: Mol Cell Biol. 2019 Aug 12;39(17):e00080-19. doi: 10.1128/MCB.00080-19 (PMC6692125; doi:10.1128/MCB.00080-19)

Sup FIG 1 MafB was specifically deleted from  $\beta$ -cells

A

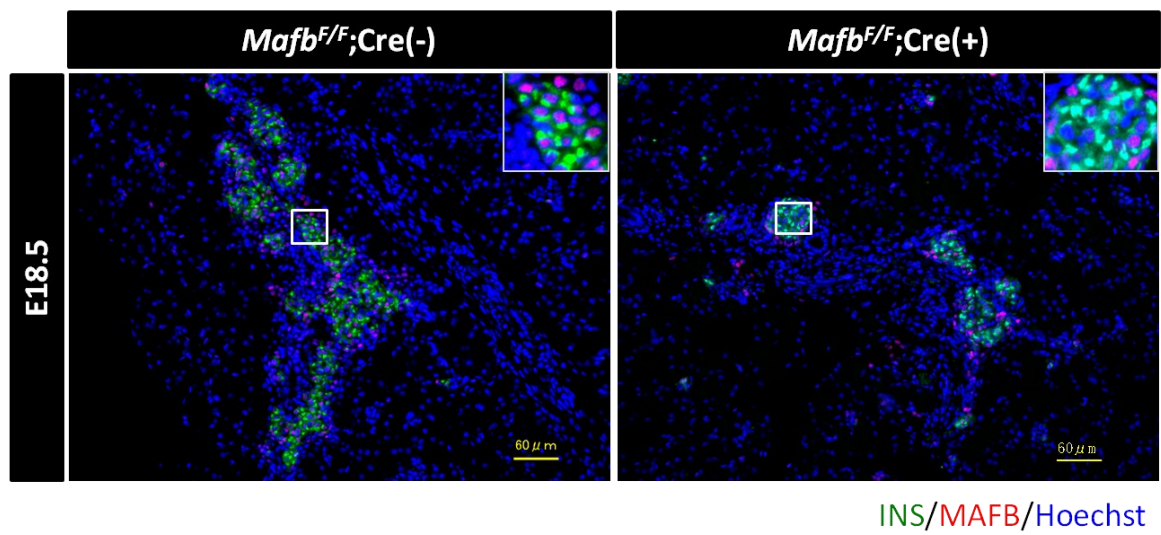

Supplement: Supplemental file 1 [file MCB.00080-19-s0001.pdf]

Sup FIG 2 MafA deletion is successful in the C57BL/6J mice strain.

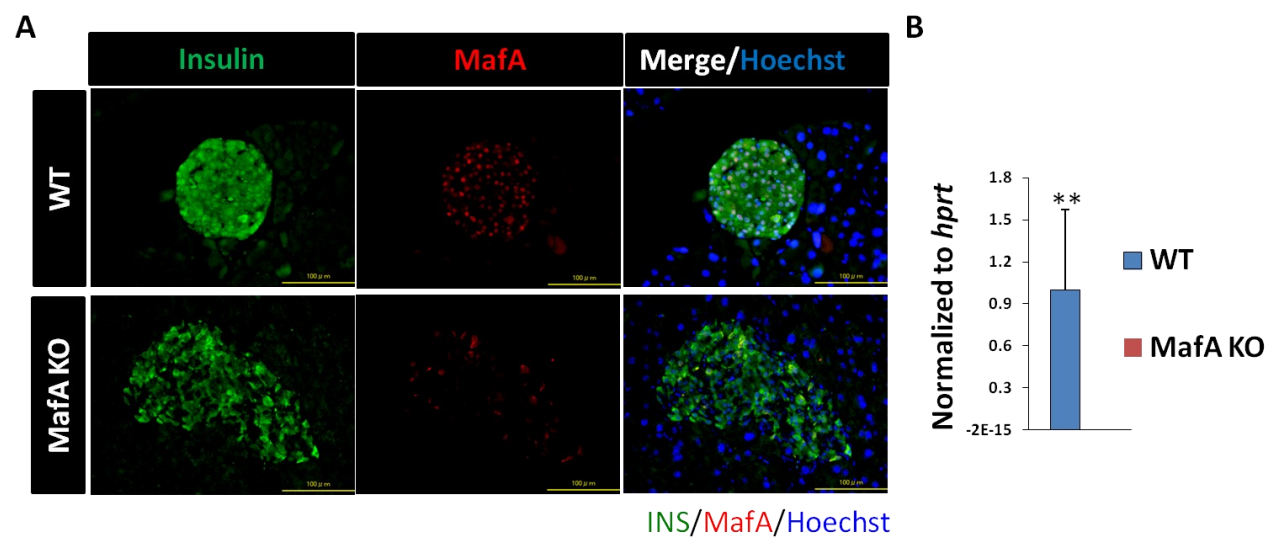

Supplement: Supplemental file 2 [file MCB.00080-19-s0002.pdf]

Sup FIG 4 Cell proliferation was not detected in all mice group 5 month after HFD treatment

A

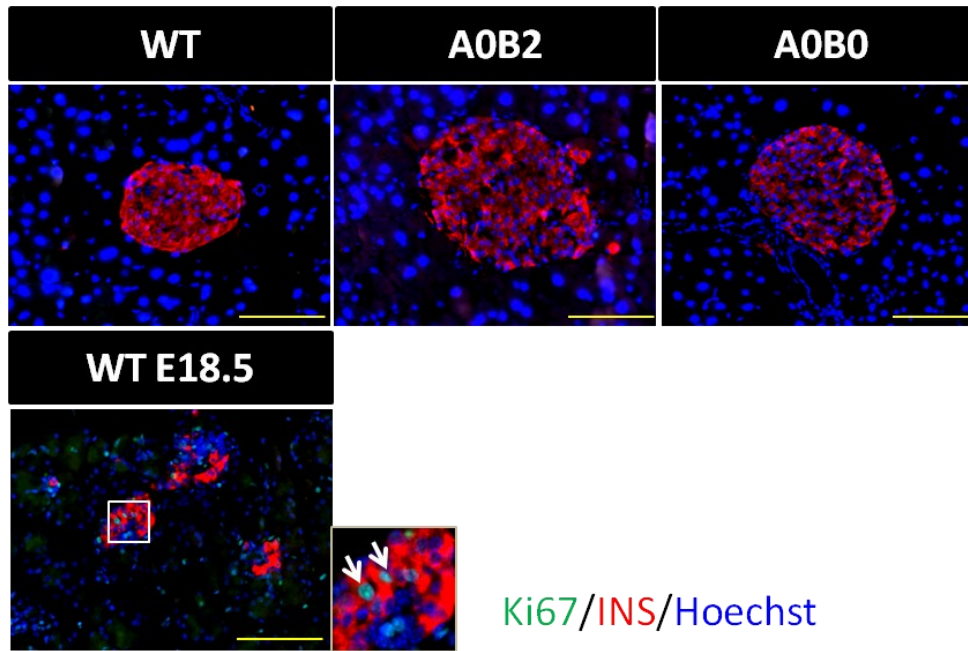

Supplement: Supplemental file 4 [file MCB.00080-19-s0004.pdf]
