# Supplementary material for: MafB Is Important for Pancreatic β-Cell Maintenance under a MafA-Deficient Condition
Source: Mol Cell Biol. 2019 Aug 12;39(17):e00080-19. doi: 10.1128/MCB.00080-19 (PMC6692125; doi:10.1128/MCB.00080-19)

Sup FIG 3 β-cell to α-cell conversion was not detected in all mice group

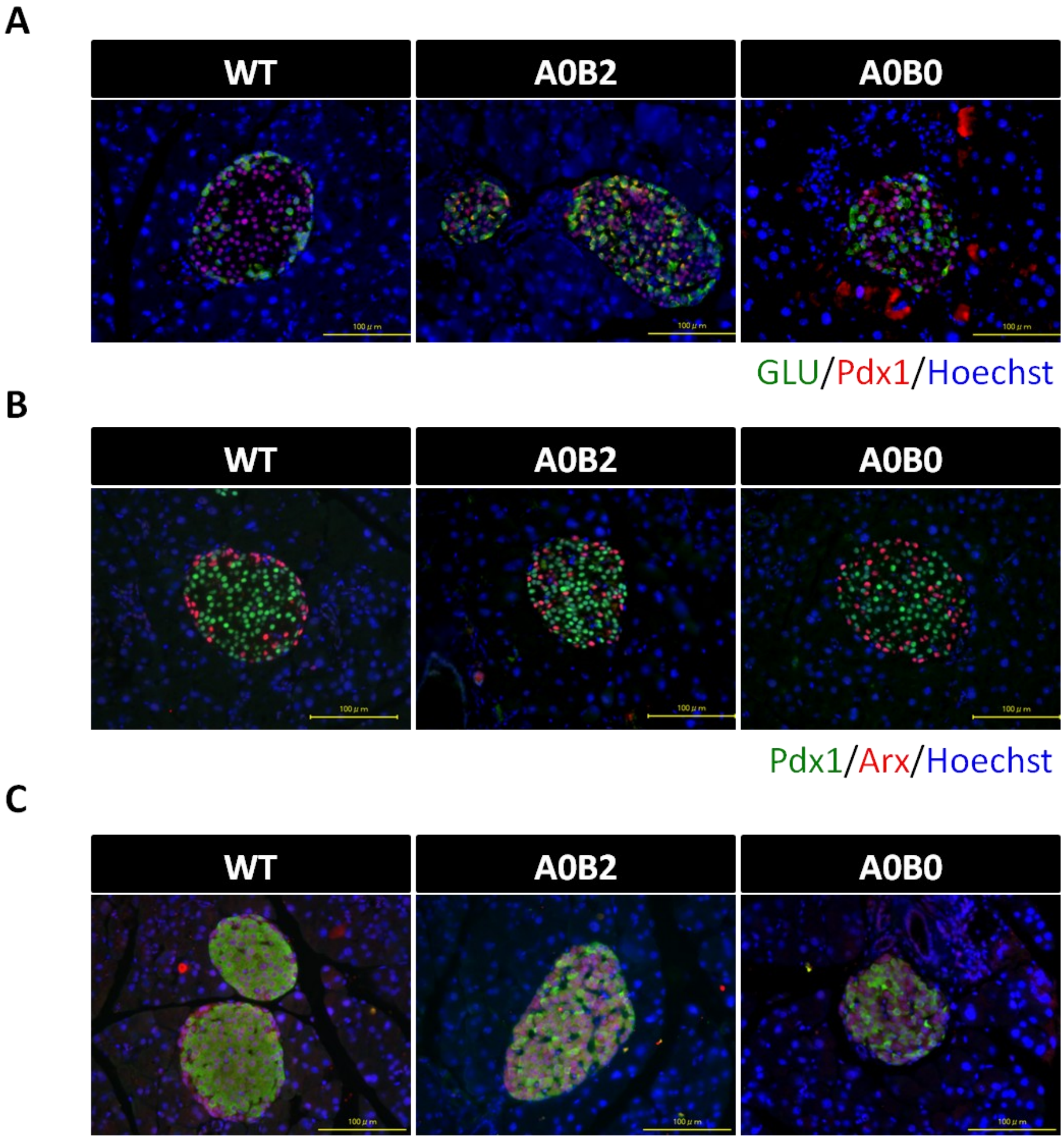

Supplement: Supplemental file 3 [file MCB.00080-19-s0003.pdf]
