# Supplementary material for: MafB Is Important for Pancreatic β-Cell Maintenance under a MafA-Deficient Condition
Source: Mol Cell Biol. 2019 Aug 12;39(17):e00080-19. doi: 10.1128/MCB.00080-19 (PMC6692125; doi:10.1128/MCB.00080-19)
Supplement: Supplemental file 5 [file MCB.00080-19-s0005.pdf]

Figure Legends

Sup FIG 1

MafB was specifically deleted from pancreatic β-cells. A) Insulin (green) and MafB (red) immunoreactivity in MafB $^{F/F}$:: Cre^{-} and MafB $^{F/F}$:: Cre^{+} mice at embryonic day 18.5 (E18.5). Scale bar, 100 μm.

Sup FIG 2

MafA deletion is successful in the C57BL/6J mice strain. A) Insulin (green) and MafA (red) immunoreactivity in WT and MafA knockout mice. Scale bar, 100 μm. **B)** *Mafa* gene expression of islets from each genotype. The amount of each transcript was normalized to the amount of the *Hprt* transcript. Data are from 4 to 6 male mice of each genotype at 9 months. *, $p < 0.05$ and **, $p < 0.01$.

Sup FIG 3

α-cell to β-cell conversion was not detected in all mice group. A) Glucagon (green) and Pdx1 (red) immunoreactivity in pancreatic islets from each genotype; **B)** Pdx1 (green) and Arx (Red) immunoreactivity in pancreatic islets from each genotype; **C)** Insulin (green) and Pdx1 (red) immunoreactivity in pancreatic islets from each genotype; 3-4 female mice from each genotype were used at 9 month old age. Scale bar, 100 μm.

Sup FIG 4

Cell proliferation was not detected in the islets of all mice group 5 months after HFD treatment. A) Ki67 (green) and insulin (red) costaining of islet from WT mice at E18.5. **B)** Ki67 (green) and insulin (red) costaining of islet from each genotype after HFD treatment. One male mouse from each genotype was used after 5-month HFD treatment. Scale bar, 100 μm.
